# The Relationship between Urinary Incontinence, Osteoarthritis, and Musculoskeletal System Disorders

**DOI:** 10.3390/jcm13082272

**Published:** 2024-04-14

**Authors:** Nursanem Celik, Suleyman Celik, Zuleyha Seyhan, Muhammed Furkan Dasdelen, Furkan Almas, Selami Albayrak, Rahim Horuz, Pilar Laguna, Jean de la Rosette, Mehmet Kocak

**Affiliations:** 1 School of Medicine, Istanbul Medipol University, 34083 Istanbul, Türkiye; nursanem.celik@std.medipol.edu.tr (N.C.); salbayrak@medipol.edu.tr (S.A.); rhoruz@medipol.edu.tr (R.H.); 2 International School of Medicine, Istanbul Medipol University, 34810 Istanbul, Türkiye; zuleyha.seyhan@std.medipol.edu.tr (Z.S.); muhammed.dasdelen@std.medipol.edu.tr (M.F.D.); furkan.almas@std.medipol.edu.tr (F.A.); plaguna@medipol.edu.tr (P.L.); j.j.delarosette@gmail.com (J.d.l.R.); mehmetkocak@medipol.edu.tr (M.K.); 3 Department of Urology, Istanbul Medipol University, 34083 Istanbul, Türkiye; 4 Department of Biostatistics and Medical Informatics, Istanbul Medipol University, 34083 Istanbul, Türkiye

**Keywords:** urinary incontinence, back musculoskeletal system disorders, osteoarthritis, chronic pain, mobility impairment

## Abstract

**Background/Objectives**: Urinary incontinence diminishes quality of life, and its severity can be worsened by mobility impairments. This study explored the link between urinary incontinence, osteoarthritis, and back musculoskeletal system disorders, considering pain, mobility issues, and daily activity difficulties. **Methods**: This cross-sectional study included respondents aged ≥ 15 years from the 2008 Turkish Health Studies Survey (n = 13,976). We assessed self-reported urinary incontinence, daily activity, mobility impairment, pain, osteoarthritis, and musculoskeletal disorders to explore their association with urinary incontinence. Gender-specific logistic regression models included chronic conditions related to urinary incontinence. **Results**: The prevalence of urinary incontinence was higher in the participants with osteoarthritis and back musculoskeletal system problems. Among the patients with osteoarthritis, the prevalence was 25.84% in the mobility-impaired group and 10.03% in the non-impaired group. Similarly, 33.02% of those with activities of daily living (ADL) difficulties and 12.93% of those without difficulties had incontinence. The frequency of urinary incontinence increased with pain severity. According to the multivariable logistic regression analyses, the adjusted odds ratio (95% confidence interval) of urinary incontinence for osteoarthritis was 1.58 (95% CI 1.23–2.02, *p* < 0.01) for females and 2.38 (95% CI 1.62–3.49, *p* < 0.01) for males. **Conclusions**: Urinary incontinence was more common in females, increased with age, and was found to be associated with osteoarthritis and back musculoskeletal system disorders. Among the patients with osteoarthritis and back musculoskeletal system disorders, those with mobility impairment and daily activity difficulties had a higher prevalence of urinary incontinence. The patients with more severe pain had a higher frequency of urinary incontinence.

## 1. Introduction

Urinary incontinence (UI) is defined as involuntary urine loss, categorized in five major types as stress, urgency, overflow, functional, and mixed urinary incontinence.

It is a distressing, socially restricting, and embarrassing condition. It may lead to a decline in health-related quality of life (HRQoL), daily physical activity, and productivity.

The etiology of UI is not fully understood, but it is multifactorial, and it can be chronic or temporary condition that results from an underlying medical condition. Increased abdominal pressure, overactive bladder, poor detrusor contractility, and outlet obstruction are attributed UI, and it may be associated with different physiological or pathological circumstances in different gender and age sets.

The risk factors can be grouped into behavioral risk factors (lifestyle), physiological risk factors, demographic risk factors (social, economic cultural) environmental and genetic risk factors [1].

Urinary incontinence is a common health problem in the general population, with a higher incidence in women [2].

The incidence of UI is high during both pregnancy and postpartum. Furthermore, the incidence of urinary incontinence ranges from 21.3% to 40% at different stages of pregnancy, depending on the country or region. Besides, with the increase in the number of deliveries, the risk of the incontinence rises [3]. In males, prostate problems are the main factors associated with UI [4].

Increasing age is a risk factor in both genders for developing UI. Aging itself is not a cause of UI, although age-related changes in lower urinary tract function can predispose older people to UI, which is then worsened by comorbidities [2].

Overall, 11–34% of men and 13–50% of women over 60 years old suffer from urinary incontinence [5].

Recent insights, particularly regarding central bladder control and the intricacies of bladder storage and micturition functions, have unveiled a significantly more nuanced understanding of incontinence among the elderly compared to just a few years ago. Individuals up to the age of 65 with impaired bladder function typically exhibit functional defects in the bladder, bladder outlet, or pelvic floor. However, the capacity to maintain continence in old age is primarily influenced by changes in neurogenic control and the gradual weakening of compensatory mechanisms [6].

Previous studies have found associations of urinary incontinence with several chronic conditions, including type 2 diabetes mellitus, obesity, hypertension, heart failure, chronic obstructive pulmonary disease, asthma, bowel disorders, and arthritis [7].

Although urinary incontinence and its association with chronic diseases are broadly discussed in the literature, very little has been written about UI in musculoskeletal disorders.

Osteoarthritis (OA) is the most common musculoskeletal disorder, resulting from various changes in physiology, anatomy, and the biomechanics of joint cartilage. It mainly affects the knee and hip, causing pain, stiffness, swelling, and loss of normal joint function. It is more common in women and has a higher prevalence in the elderly [8]. Similar observations can be made in patients with back musculoskeletal system disorders (BMSD), including spine hernia, muscle strain, ligament sprain, myofascial pain, fibromyalgia, spinal fracture, and vertebral osteomyelitis, which may result in complaints that impair daily activity and functional capacity [9].

Both OA and BMSD may impair mobility, and the functional and mobility limitations experienced in both conditions not only worsen the quality of life, but may also lead to an increased likelihood of UI [10,11]. A limited number of studies have explored the influence of musculoskeletal system disorders and osteoarthritis on the severity of the presentation of UI [12,13]. However, the evidence provided is speculative and the knowledge gap in this area remains.

In a previous study, we suggested that OA has significant relationship with UI in both genders [14]. The primary objective of the present study was to explore the relationship between urinary incontinence, osteoarthritis, and BMSD in the Turkish population. Secondly, we aimed to study the link between urinary incontinence and conditions such as pain severity, mobility impairment, and difficulties in performing daily activities.

## 2. Materials and Methods

### 2.1. Study Population

This cross-sectional study utilized data from the 2008 Turkish Health Survey, which was conducted by the Turkish Statistical Institute (TurkStat) [15]. The survey included information from 20,624 participants and was designed to provide sufficient representation for 26 basic regions to support regional policy applications. TurkStat, following Eurostat procedures, establishes the fundamental principles governing the production and management of official statistics in Turkiye. Since 2008, TurkStat has conducted the Turkish Health Survey (THS) every two years to assess health profiles, sociodemographic characteristics, and physical health statuses. The first data made publicly available were from the survey conducted in 2008. The survey was administered through face-to-face interviews, and the responses to all the variables were self-reported.

Participants were classified into seven age groups: 15–24 years, 25–34 years, 35–44 years, 45–54 years, 55–64 years, 65–74 years, and 75 years or older. Respondents were asked about past or present experiences with long-term conditions, including asthma, chronic bronchitis, chronic obstructive pulmonary disease, myocardial infarction, coronary heart diseases, heart failure, hypertension, stroke, osteoarthritis, rheumatoid arthritis, diabetes mellitus (DM), cancer, and depression.

In the survey, education levels were assessed across various tiers, including categories such as illiterate, no formal education, primary school, primary education, secondary school, high school, university degree, and master’s/doctorate equivalent. Marital status was classified as married, divorced, widowed or single. Area of residence (rural or urban) formed another independent variable.

TurkStat obtained ethical clearance and informed consent from each respondent. Study outcomes, accessible through TurkStat, were anonymized for identity protection. Data collection used a two-stage cluster sampling method, stratified by rural and urban areas [14].

### 2.2. Measures

We evaluated patients aged ≥ 15 years (n = 13,976). We initially identified predefined risk factors and causes of urinary incontinence, which we then incorporated as adjustment variables in our logistic regression analysis.

Current health status of the participants was assessed by inquiring about chronic diseases in the survey, with response options including (1) Yes, (2) No, (3) Don’t know, or (4) Refusal. Options 3 and 4 were treated as missing values, leading to exclusion from our analysis.

In this study, urinary incontinence was determined solely based on self-reported responses, specifically by asking about the experience of urinary leakage, irrespective of the type of incontinence. These reports of experiencing leakage were utilized to label individuals as normal or incontinent.

Yes or No answers to ‘Do you have following condition?’ were evaluated for OA and BMSD, and then they were analyzed accordingly. This same categorization method was applied to other chronic diseases utilized in our study.

Pain was assessed based on respondents’ answers to questions that inquired about the severity of physical discomfort and pain intensity, utilizing a scale that included options such as none, mild, moderate, severe, and extreme pain. Responses were collected as self-reported, without confirmation by doctor examination and consultation. Any participants with missing values for these pain assessment questions were also excluded from our study group. Next, each category of pain severity was coded as patient with no pain, mild pain, moderate pain, or severe/extreme pain. Subsequently, UI prevalence was calculated in each group for comparison. Pain medication was assessed with the answer to the question, ‘During the past two weeks have you used any medication for this condition,’ which was applied to OA, BMSD, pain in the joints, and other pains.

We used the formula derived from the International Physical Activity Questionnaire (IPAQ) to assess exercise, categorizing it as high-intensity (3 days vigorous or 5 days moderate per week), sedentary (no exercise), or low-intensity (in-between) [16]. Using these three categories, urinary incontinence and musculoskeletal system disorders were evaluated.

In our study, we categorized education level as high-school degree and higher or secondary level vs. less, and marital status as single vs. married. Rural or urban status was evaluated as a control variable.

Anxiety and depression were assessed under the chronic condition question, with the answers to the question of ‘Do you have any of the following diseases?’ linked to anxiety and depression. Answers were self-reported and without confirmation by healthcare providers.

Cardiac diseases, including hypertension, coronary heart diseases, myocardial infarction, and chronic heart failure, evaluated under chronic conditions question. Individuals with any of these conditions were categorized as having cardiac diseases and analyzed accordingly.

We assessed mobility impairment based on respondents’ answers to a combination of two questions from the survey: (1) ‘Do you experience any difficulties walking 500 m without assistance,’ and (2) ‘Do you have any difficulties climbing 10 steps upstairs?’ Respondents who reported difficulties in response to either of these questions were categorized as mobility-impaired patients, and others were classified as normal [11].

We determined the functional status of individuals based on a sequence of questions that assessed any reported difficulties in activities of daily living (ADL) and instrumental activities of daily living (IADL), as established in previous studies. For the ADL assessment, we used the Katz index of independence, which includes questions about the following activities: feeding oneself, dressing, using the toilet, bathing, and standing from a bed or chair. Respondents who reported difficulties in any of these activities were classified as ‘ADL-impaired.’ To assess IADL impairment, we employed the Brody Instrumental Activities of Daily Living scale, which included questions about preparing meals, performing light or heavy housework, managing money, and shopping. Any reported difficulties in these actions were recognized as ‘IADL-impaired’ [11,17]. Individuals who refused to answer or lacked knowledge regarding pain, mobility, or functional status questions were considered as having missing values and were consequently excluded from our analysis.

### 2.3. Statistical Analysis

Patient sociodemographic and disease-related characteristics were described using frequencies and percentages for categorical variables and mean and standard deviation for continuous variables. As primary models, multivariable logistic regression models were constructed to investigate the association of urinary incontinence with chronic OA and BMSD, controlling for potential confounding variables (diabetes, rheumatoid arthritis, asthma, COPD, cardiac diseases, anxiety, depression, and stroke). Age, marital, education, and residency status were also included in the primary model as adjusting variables. These primary models were performed separately for males and females.

In addition, in separate multivariable logistic regression models, OA and BMSD were considered as outcome variables, and their association with age, gender, mobility impairment, IADL difficulties, and extreme, severe, moderate, and mild pain were investigated.

Statistical significance was determined at a Type-1 error rate of 0.05. The multivariate logistic regression analyses were performed using The Statsmodels library in Python 3.9. GraphPad Prism 10.2.2 was used for data visualization and related statistical analyses. Certain icons in the graphical abstract were created with the assistance of DALL-E v3 (https://chat.openai.com/ accessed on 28 February 2024).

## 3. Results

**Figure 1:**
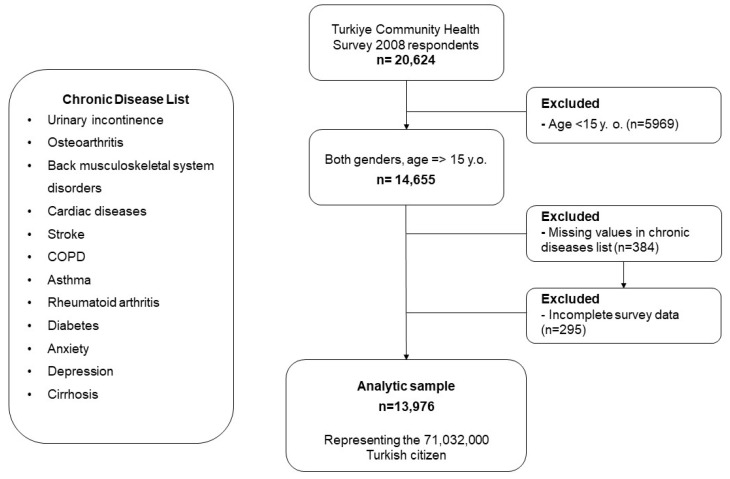
Flow diagram of the population selection according to study criteria.

Among the 13,976 participants, 189 (2.98%) men and 445 (5.82%) women reported experiencing UI (Figure 1). Roughly 17% of those who reported having OA and 12% of those who reported having BMSD experienced UI. Compared to those who did not have UI, the participants with UI were significantly older, experienced more severe pain, and had impaired ADL and mobility. Regarding residency status, there was a greater prevalence of UI (6.44%) among the individuals residing in rural areas compared to those in urban areas (3.70%) (Table 1).

**Figure 2:**
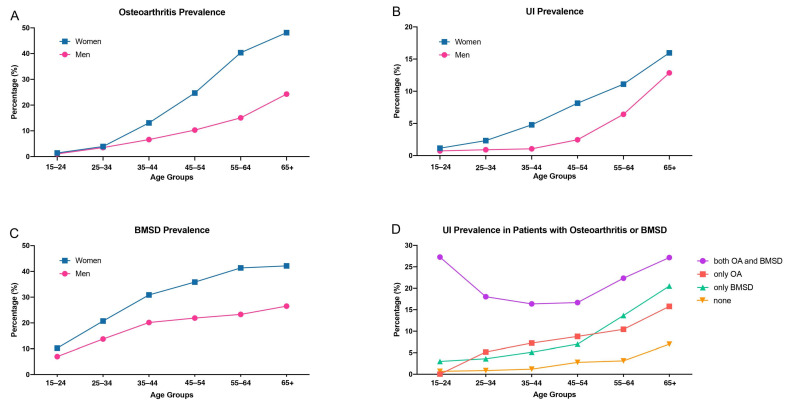
Abbreviations: UI; urinary incontinence, OA; osteoarthritis, BMSD; back musculoskeletal system disorders. Comparison of UI, osteoarthritis, and BMSD prevalence over ages between males and females (**A**–**C**). Frequency of UI in patients with BMSD or OA only, both OA and BMSD concurrently, and patients without OA or BMSD (**D**).

The prevalence of OA, UI, and BMSD all exhibited a common trend, with higher rates observed in women and an increasing likelihood associated with advancing age (Figure 2A–C). The prevalence of other chronic conditions showed the same inclination (Supplementary Figure S2).

When comparing individuals with no musculoskeletal system problems, those who reported both BMSD and OA exhibited the highest frequency of incontinence, while the individuals with either OA or BMSD alone also showed a notably higher frequency of incontinence in all the age groups (Figure 2D).

Gender-specific logistic regression models for UI are displayed in Table 2. In these models, after adjusting for known confounding factors, OA and BMSD were associated with UI in both men and women. Anxiety, diabetes, rheumatoid arthritis, cardiac diseases, and age were also significantly associated with UI in both genders. In contrast, COPD and marital status were not significantly associated with UI in either gender. However, there were slight differences between the gender-specific models; stroke and depression were significantly associated with UI only in the females, whereas asthma was associated only in the males. Living in a rural area was associated with UI in the males but not in the females. Secondary-level of education was related to UI in the females with ORs 0.6 (95% CI 0.42–0.87, *p* < 0.01), but not higher education. There was no association between education level and UI in the males (Table 2).

We estimated the components of OA and BMSD in separate logistic regression models. Experiencing mild, moderate, severe, and extreme pain, suffering from mobility impairment and IADL difficulties, increasing age, and female gender were associated with both OA and BMSD. Extreme pain was the strongest predictor of OA and BMSD with ORs 5.97 (95% CI 4.12–8.65, *p* < 0.01) and 5.53 (95% CI 3.98–7.69, *p* < 0.01), respectively (Supplementary Figure S1).

**Figure 3:**
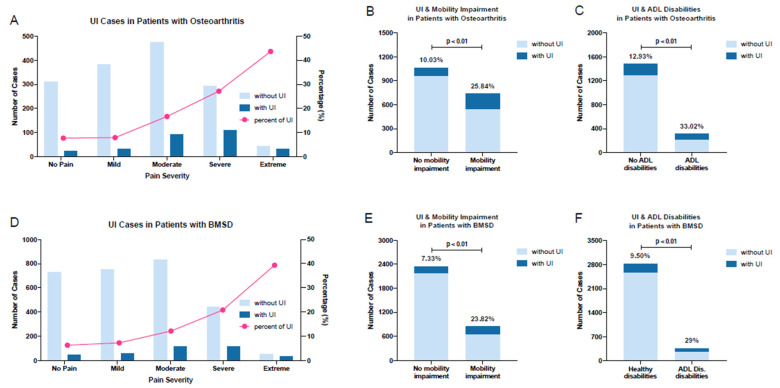
Abbreviations: UI; urinary incontinence, OA; osteoarthritis, BMSD; back musculoskeletal system disorders, ADL; activities of daily living. Frequency and number of reported UI in osteoarthritis patients with different pain levels (**A**); UI and mobility impairment in patients with OA (**B**); UI and ADL disabilities in patients with OA (**C**); frequency and number of reported UI in BMSD patients with different pain levels (**D**); UI and mobility impairment in patients with BMSD (**E**); UI and ADL disabilities in patients with BMSD (**F**).

In the patients with OA, the frequency of UI was 25.84% in the individuals with mobility impairments and 10.03% in those without impairment (*p* < 0.01) (Figure 3B). Furthermore, the frequency of UI was 33.02% in those with ADL difficulties and 12.93% in those without difficulties (*p* < 0.01) (Figure 3C). Likewise, when we analyzed the patients with BMSD, we observed that the frequency of UI was 23.82% in the individuals with mobility impairments and 7.33% in those without impairment (*p* < 0.01) (Figure 3E). It was 29% in those with ADL difficulties and 9.50% in those without difficulties (*p* < 0.01) (Figure 3F). Pain severity was another factor influencing UI. The prevalence of UI in the OA and BMSD patients increased with greater pain severity (Figure 3A,D).

## 4. Discussion

The prevalence of UI in the present study was 2.97% for men, and it was 5.81% for women, both of which are similar to the prevalence reported in other population-based studies [18]. The key finding of the current study was a strong association between UI, BMSD, OA, pain severity, mobility impairment, and difficulty in daily activities. Among the patients with OA and BMSD, the higher the pain severity, the higher the likelihood of experiencing UI. In the individuals with mobility impairment and ADL difficulties, there was a higher prevalence of UI. As expected, females had a higher frequency of UI. It has been suggested that gynecological and obstetric events may contribute to this higher prevalence [19]. Additionally, we observed that OA and BMSD were more prevalent in females, which is possibly explained by anatomical, hormonal, and biological differences [20].

There is a body of research focusing on the prevalence of chronic conditions. Data from the Canadian Community Health Survey in 2013–2014 (n = 60,168) showed that 45.8% of females reported having at least one chronic condition and that 20.2% reported two or more [7]. The most prevalent chronic conditions were arthritis (22.8%) and hypertension (20.7%). We found a lower rate of OA in our study (12.92%) than that reported by Natalie et al. [7]. This difference may be attributed to Natalie et al.’s sample and analysis, which included all types of arthritis and only women. Conversely, the frequency of BMSD in our sample was 22.86%, in line with previous studies after age and gender stratification [13,21].

The literature contains numerous studies indicating an association between chronic conditions and UI. In one particular study, Cynthia et al. [22] concluded that increasing body mass index (BMI), lung disease, cardiac diseases, hypertension, stroke, arthritis, and diabetes were associated with UI. Similarly, in the current study, OA and BMSD were strong predictors of UI in both genders, according to our comprehensive regression model, with the presence of diabetes, asthma, cardiac problems, rheumatoid arthritis, stroke, and anxiety/depression as independent variables. In our analysis, stroke was not significant in the male model, along with other chronic conditions. While asthma was only found to be significant in the male model, COPD was found to be an insignificant predictor of UI in both males and females. The conditions with the highest odds for UI in the males were anxiety, OA, and BMSD, whereas in the females, they were BMSD, anxiety, and depression.

Individuals with back pain or OA face a considerably increased risk of UI. This increased risk has been attributed to the direct effects of the skeletal disease or of the pain medication prescribed [23] and to mobility impairment delaying/making difficult the taking off pants [16]. The risk is present in both male and female genders and increases with age [10,18,21]. 

The present study stands out for its separate analysis of both males and females, in contrast to previous research primarily focusing on older women with UI. Additionally, we included a wider age spectrum, encompassing both men and women aged 15 years and older. Our analysis revealed significantly higher rates of urinary incontinence among patients with osteoarthritis and BMSD across all age groups. In support of previous studies, we concluded that the higher the severity of pain, the greater the likelihood of urinary incontinence.

Previous studies reported that mobility impairment and falls were associated with UI. Factors such as walking speed, duration of raising, and gait balance in women aged between 75 and 85 reveal a UI frequency of 42% and an association between impaired mobility and urge urinary incontinence, but not stress UI [24]. Our data align with these findings, demonstrating that patients with OA and mobility impairments exhibit a substantially higher prevalence of urinary incontinence (25.84%) compared to OA patients without such impairments (10.02%). It can be speculated that patients with mobility issues may struggle to reach the toilet on time and face difficulties in undressing in the bathroom.

One particular study [25] analyzed individuals with hip fractures, and the authors found that dependence on wheelchairs and other ambulation devices increased the risk of UI. Additionally, other studies pointed out that different functional impairments (ADL, IADL) may be related to becoming incontinent [10]. Our results contributed to the association of ADL difficulties with incontinence. The frequency of UI was higher in the patients with impaired ADL (33.02% vs. 12.93%).

There are several limitations in our study, mostly derived from its general design as a population-based global health evaluation and from its cross-sectional design. The general character of the survey did not involve UI distinction and characterization. Therefore, no information on the distinction between urge incontinence (overactive bladder) and stress incontinence was possible. While they frequently overlap, these conditions have different causes.

Furthermore, while suggesting that mobility impairments resulting from OA and BMSD are risk factors for UI, we are unable to distinguish whether this association primarily involves functional UI or other types. Furthermore, we lack insight into whether UI improves after recovery from these mobility impairments. Given these gaps in knowledge, future research in the form of longitudinal studies investigating the long-term effects of mobility impairment on UI, as well as the potential improvement in UI after recovery, is imperative.

In addition to the lack of UI characterization, UI was self-reported, severity was not quantified, and potential surgical or iatrogenic causality was not discerned. Furthermore, the impact of UI on quality of life was not investigated by proper patient-reported-outcome questionnaires. To address those limitations, future studies should adopt a more comprehensive and sophisticated approach to investigating the relationship between mobility impairments and UI.

Lastly, the lack of some variables (e.g., BMI could not be calculated due to the absence of weights and heights) prevented us from controlling for some potential conditions associated with sporadic UI, and the cross-sectional design did not allow us to determine the possible causes of sporadic UI (e.g., urinary tract infection). Despite these limitations, this study is the first and largest population-based study in Turkiye to investigate the relationship between UI, OA, and BMSD. It represents a snapshot of the Turkish population, mirroring data reported in developed countries, and ultimately identifies musculoskeletal system problems as potential treatment targets that could potentially improve UI. 

## 5. Conclusions

In summary, our study contributes significant insights into the relationship between urinary incontinence (UI) and musculoskeletal disorders, particularly osteoarthritis (OA) and back muscle skeletal disorders (BMSD), among both men and women in Turkiye. Our findings reveal a higher prevalence of UI in females and demonstrate strong associations between UI and factors such as pain severity, mobility impairment, and difficulties in activities of daily living (ADL).

By highlighting the elevated risk of UI among individuals with OA and BMSD, as well as the impact of pain severity, mobility limitations, and ADL impairments on UI prevalence, our study underscores the importance of comprehensive assessment and targeted interventions in managing UI in this population.

Despite limitations inherent to our study design, including its cross-sectional nature and the lack of detailed characterization of UI subtypes, our findings provide a foundational understanding of the epidemiology of UI in Turkiye. Future research should aim to address these limitations through longitudinal data collection and standardized measures to assess UI severity and its impact on quality of life.

Overall, our study underscores the significance of considering musculoskeletal health in the management of UI and suggests avenues for future research and interventions aimed at improving the well-being and quality of life of individuals affected by these conditions.

## Figures and Tables

**Table 1:** Demographics of the participants.

Variables	Overall (n = 13,976)	Percentage %	With UI (n = 634)	Percentage %
**Age**				
15–24	2787	19.94	27	0.96
25–34	3188	22.81	54	1.69
35–44	2772	19.83	88	3.17
45–54	2303	16.48	125	5.42
55–64	1512	10.82	134	8.86
65+	1414	10.12	206	14.56
**Gender**				
Male	6331	45.30	189	2.98
Female	7645	54.70	445	5.82
**Exercise**				
Low intensity	6254	44.75	281	4.49
High intensity	2467	17.65	107	4.33
Non-exercising	5255	37.60	246	4.68
**Pain-severity**				
No pain	7662	54.82	115	1.50
Mild	2938	21.02	112	3.81
Moderate	2176	15.57	186	8.54
Severe	1032	7.38	171	16.56
Extreme	168	1.20	50	29.76
**Residency**				
Rural	4239	30.33	273	6.44
Urban	9737	69.66	361	3.70
**Marital Status**				
Single	4209	30.12	162	3.85
Married	9767	69.88	472	4.83
**Education**				
Elementary level	6503	46.53	356	5.47
Secondary level	4571	32.71	70	1.53
Higher education	2902	20.76	208	7.16
**Osteoarthritis**	1808	12.94	299	16.53
**BMSD**	3196	22.86	374	11.70
**Mobility Impairment**	1785	12.76	301	16.86
**Functional Disability**	2523	18.03	363	14.38
**ADL Disability**	705	5.03	155	21.99
**IADL Disability**	3935	28.14	410	10.42
**Pain Medication**	3373	24.13	312	9.25

Abbreviations: BMSD: back musculoskeletal system disorders; ADL: activities of daily living; IADL: instrumental activities of daily living. Characteristics of study population (n = 13,976).

**Table 2:** Adjusted odd ratios for urinary incontinence.

	Female UI OR (95% Cl)	Male UI OR (95% Cl)
	(Model I)	(Model II)
**Anxiety**		
No	Reference	Reference
Yes	2.25 (1.54–3.28)	2.98 (1.36–6.54)
	*p* < 0.001	*p* < 0.01
**Osteoarthritis**		
No Yes	Reference 1.58 (1.23–2.02)	Reference 2.38 (1.62–3.49)
	*p* < 0.001	*p* < 0.001
**BMSD**		
No	Reference	Reference
Yes	2.55 (2.04–3.20)	2.00 (1.41–2.82)
	*p* < 0.001	*p* < 0.001
**Asthma**		
No	Reference	Reference
Yes	1.14 (0.82–1.59)	1.84 (1.07–3.17)
	*p* = 0.4	*p* = 0.02
**Diabetes**		
No	Reference	Reference
Yes	1.52 (1.15–2.05)	1.82 (1.18–2.80)
	*p* < 0.001	*p* < 0.01
**Rheumatoid arthritis**		
No	Reference	Reference
Yes	1.68 (1.32–2.14)	1.68 (1.16–2.44)
	*p* < 0.001	*p* < 0.01
**Cardiac diseases**		
No	Reference	Reference
Yes	1.60 (1.25–2.04)	1.48 (1.04–2.11)
	*p* < 0.001	*p* = 0.02
**Stroke**		
No	Reference	Reference
Yes	1.89 (1.13–3.15)	1.39 (0.57–3.39)
	*p* = 0.01	*p* = 0.46
**Depression**		
No	Reference	Reference
Yes	2.12 (1.55–2.89)	1.28 (0.54–3.04)
	*p* < 0.001	*p* = 0.57
**Education**		
Elementary level	Reference	Reference
Secondary level	0.6 (0.42–0.87)	0.72 (0.46–1.14)
	*p* < 0.01	*p* = 0.17
**Higher education**	0.96 (0.76–1.22)	1.08 (0.72–1.63)
	*p* = 0.78	*p* = 0.66
**Marital status**		
Single	Reference	Reference
Married	0.96 (0.76–1.22)	0.75 (0.47–1.20)
	*p* = 0.76	*p* = 0.24
**COPD**		
No	Reference	Reference
Yes	1.50 (0.96–2.35)	0.74 (0.36–1.55)
	*p* = 0.07	*p* = 0.43
**Residency status**		
Rural	Reference	Reference
Urban	0.88 (0.71–1.10)	0.51 (0.37–0.70)
	*p* = 0.28	*p* < 0.001
**Age**		
Normal	Reference	Reference
Elderly	1.22 (1.12–1.34)	1.56 (1.37–2.78)
	*p* < 0.001	*p* < 0.001

Abbreviations: OR: odds ratio; Cl: confidence interval; UI: urinary incontinence; BMSD: back musculoskeletal system disorders; COPD: chronic obstructive pulmonary diseases. Note: In model I, odds for UI were calculated in females. In model II, odds for UI were calculated in males.

## Data Availability

The data used in this study were taken from the Turkish Statistical Institute (TurkStat), but the availability of these data is limited due to certain constraints. The data were authorized specifically for this study and are not publicly available. However, the authors can provide the data upon reasonable request, with permission from TurkStat and the Regional Ethical Committee.

## Supplementary Materials

The following supporting information can be downloaded at: https://www.mdpi.com/article/10.3390/jcm13082272/s1. Supplementary Figure S1. Multiple variable logistic regression models for osteoarthritis and back musculoskeletal system disorders (BMSD). Mild, moderate, severe, and extreme pain, age, gender, mobility, and functional impairment were evaluated in these models. Supplementary Figure S2. Prevalence of chronic conditions by age category. DM; diabetes mellitus, COPD; chronic obstructive pulmonary disease, UI; urinary incontinence, MI; myocardial infarction.
