# Supplementary material for: The Relationship between Urinary Incontinence, Osteoarthritis, and Musculoskeletal System Disorders
Source: J Clin Med. 2024 Apr 14;13(8):2272. doi: 10.3390/jcm13082272 (PMC11050964; doi:10.3390/jcm13082272)
Supplement: Supplementary file 1 [file jcm-13-02272-s001.zip › jcm-2923151-supplementary.pdf]

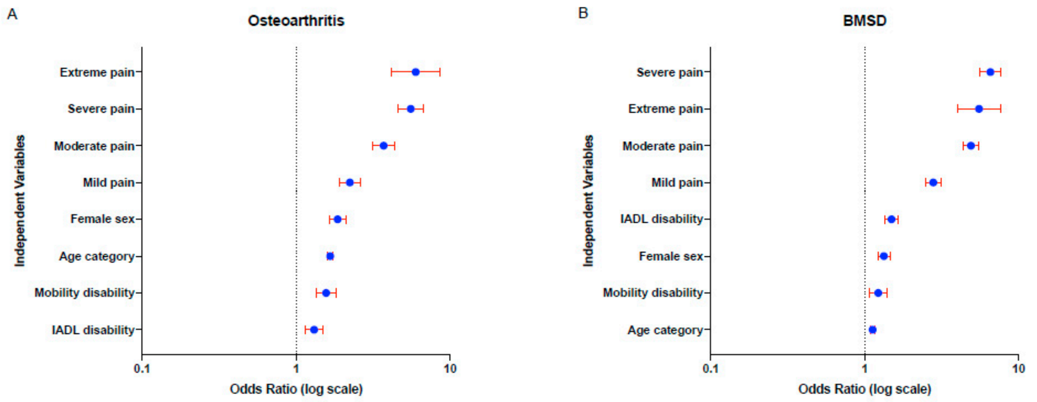

Supplementary Figure S1. Multiple variable logistic regression models for osteoarthritis and back musculoskeletal system disorders (BMSD). Mild, moderate, severe, and extreme pain; age, gender, mobility, and functional impairment were evaluated in these models.

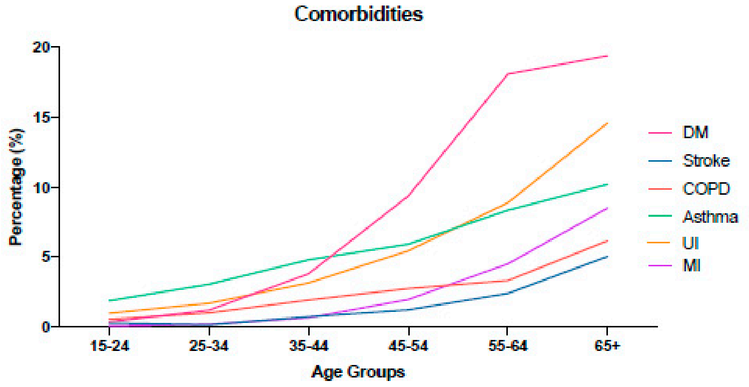

Supplementary Figure S2. Prevalence of chronic conditions by age categories. DM; diabetes mellitus, COPD; chronic obstructive pulmonary disease, UI; urinary incontinence, MI; myocardial infarction
